# Cryo-EM structure of an ATTRwt amyloid fibril from systemic non-hereditary transthyretin amyloidosis

**DOI:** 10.1038/s41467-022-33591-4

**Published:** 2022-10-27

**Authors:** Maximilian Steinebrei, Juliane Gottwald, Julian Baur, Christoph Röcken, Ute Hegenbart, Stefan Schönland, Matthias Schmidt

**Affiliations:** 1 Institute of Protein Biochemistry, Ulm University, Helmholtzstrasse 8/1, Ulm, D-89081 Germany; 2 Department of Pathology, Christian-Albrechts-University Kiel, University Hospital Schleswig-Holstein, Arnold-Heller-Str. 3, Building U33, Kiel, D-24105 Germany; 3 Medical Department V, Amyloidosis Center, Heidelberg, University Hospital Heidelberg, Im Neuenheimer Feld 400, Heidelberg, D-69120 Germany

**Keywords:** Cryoelectron microscopy, Proteins, Protein aggregation

## Abstract

Wild type transthyretin-derived amyloid (ATTRwt) is the major component of non-hereditary transthyretin amyloidosis. Its accumulation in the heart of elderly patients is life threatening. A variety of genetic variants of transthyretin can lead to hereditary transthyretin amyloidosis, which shows different clinical symptoms, like age of onset and pattern of organ involvement. However, in the case of non-hereditary transthyretin amyloidosis ATTRwt fibril deposits are located primarily in heart tissue. In this structural study we analyzed ATTRwt amyloid fibrils from the heart of a patient with non-hereditary transthyretin amyloidosis. We present a 2.78 Å reconstructed density map of these ATTRwt fibrils using cryo electron microscopy and compare it with previously published V30M variants of ATTR fibrils extracted from heart and eye of different patients. All structures show a remarkably similar spearhead like shape in their cross section, formed by the same N- and C-terminal fragments of transthyretin with some minor differences. This demonstrates common features for ATTR fibrils despite differences in mutations and patients.

## Abstract (web summary)

This manuscript reports the structure of a pathologically relevant wild type ATTR amyloid fibril from systemic non-hereditary transthyretin amyloidosis. The comparison of the wild type ATTR fibril with two previous published ex vivo V30M ATTR fibrils highlighted numerous similarities between these different reconstructions, pointing to a common underlying structure for ATTR fibrils despite coming from different mutants and patients.

## Introduction

Non-hereditary ATTR amyloidosis is an aging-associated disease that arises from the formation of amyloid fibrils from misfolded wild type transthyretin (TTR) protein. The disease is estimated to affect over 25% of the male population over the age of 80^1^. The relevance of disease, with today’s aging society has never been higher and without treatment, ATTRwt amyloidosis is a fatal disease^2^. In ATTRwt amyloidosis, amyloid deposits occur mainly in heart tissue of men, where they cause severe cardiomyopathy^1,3^. Symptoms include cardiac failure, conduction disturbances, and arrhythmias^4^. Patients of ATTRwt are usually over the age of 60 years, normally in the seventh or eighth decade of life^5,6^. Approximately 50% of ATTRwt amyloidosis patients experienced carpal tunnel syndrome, due to ATTRwt amyloid fibrils deposit in the flexor retinaculum and tenosynovial tissue, 5 to 10 years before clinical manifestation of cardiac amyloidosis^2,7,8^.

TTR is mainly expressed in the liver and in the choroid plexus^9,10^. The protein is a 55 kDa tetramer that consists of four identical 127 residue protomers^11^. In order to form amyloid fibrils, the tetramer must dissociate and unfold such that the unfolded polypeptide chains are able to reassemble into amyloid. Therapeutic treatment with tafamidis meglumine (Fx-1006A) is used to stabilize TTR tetramers. Though the substance does not eliminate the existing amyloid fibril deposits, it reduces the progression of fibril formation, thus preventing the aggravation of symptoms^12^.

Some mutations in the coding gene, i.e. *TTR*, cause hereditary ATTR (ATTRv) amyloidosis, in which the TTR tetramer is more prone to dissociate, thus leading to an earlier onset of disease^13^. A common point mutation is V30M that causes hotspots of hereditary ATTR amyloidosis in Portugal, Japan, Sweden, and other countries^14^.

Symptoms of hereditary ATTRV30M amyloidosis can start with varying onset of disease. Patients with an early age of onset usually suffer from peripheral polyneuropathy or involvement of autonomous nervous system and gut, whereas patients with a late onset suffer more often from amyloid cardiomyopathy^15,16^. Amyloid deposits in the heart can be found in ATTRwt and ATTRv cases, but involvement of the peripheral nerves, gut, kidneys and the eye are only found in cases of ATTRv amyloidosis.

One of two phenotypes of fibril composition (type A or type B) are present in ATTR amyloidosis. A single patient usually only shows one of the phenotypes^16^. Type A ATTR fibrils contain fragmented ATTR monomers as well as full-length monomers. Type B fibrils are composed of only full-length ATTR monomers^17^. Type A fibrils are found for example in Swedish ATTRV30M patients suffering from ATTRv as well as in patients of ATTRwt amyloidosis. These amyloid deposits show a low affinity to bind Congo red and when imaged using electron microscopic (EM) methods, fibrils present themselves as relatively short, tightly packed, and haphazardly arranged^16,18,19^.

Here we used cryo electron microscopy (cryo-EM) to determine the molecular structure of a human ex vivo ATTRwt amyloid fibril. We compared the structure of the wild type fibril with two previously published structures of ex vivo ATTR amyloid fibrils from patients carrying the V30M mutation^20,21^. We demonstrate that all three fibrils show similar structural features.

## Results

### Cardiac pathology of ATTRwt amyloidosis

**Fig. 1:**
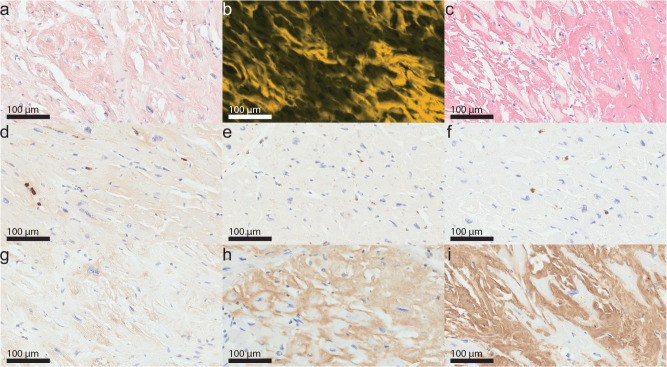
Congo red and immunostaining of ATTR deposits of heart tissue. Congo red staining in bright light (**a**) and in fluorescence light (**b**) showed massive deposits of amyloid in the heart accounting for 66% of the cross-sectional area. The amyloid deposits were immunoreactive for transthyretin (**c**) and interspersed with scanty CD3-positive T lymphocytes (**d**), CD68-positive macrophages (**e**) and occasionally myeloperoxidase-immunoreactive neutrophils (**f**). The density of inflammatory cells was not above reference values. Caspase 3-positive myocytes (**g**) were absent, but amyloid deposits reacted with the anti-complement C3 antibody (**h**) and anti-complement component C9 antibody (**i**) in the overall tissue section.

The here presented heart sample originated from an explanted heart of a male ATTRwt patient. The patient was diagnosed with ATTRwt amyloidosis 5 years prior to heart transplantation. Until heart transplantation no disease specific drugs were used. Immunohistochemistry of a formalin-fixed and paraffin-embedded heart sample showed massive interstitial amyloid deposits, which did not only encompass myocytes, but disrupted the tissue structure. Amyloid deposits accounted for 66% of the cross sectional Congo red-stained area (Fig. 1a, b), showing a very high amyloid load. They were immunoreactive for ATTR (Fig. 1c). The presence and amount of inflammation in endomyocardial biopsies was assessed by immunostaining directed against T lymphocytes (anti CD3-immunostaining), macrophages (anti-CD68-immunostaining), and neutrophils (anti-myeloperoxidase-immunostaining). However, the number of inflammatory cells in the cardiac tissue did not exceed the reference values for T-lymphocytes (>7 CD3-positive cells/mm^2^) or macrophages (>35 CD68-positive cells/mm²) (Fig. 1d, e)^22,23^. Myeloperoxidase-immunoreactive neutrophils were occasionally noted, usually within blood vessels as part of the normal blood cells (Fig. 1f). In addition, we tested for signs of single cell death, i.e., apoptosis (caspase 3) and single cell necrosis (complement 9; C9). Caspase 3-immunoreactive apoptosis (Fig. 1g) or C9-positive single cell necrosis of myocytes was not found. However, the amyloid deposits were strongly immunoreactive for C9. C9 is part of the terminal complement pathway and together with other complement components it forms a lytic pore, called the membrane attack complex^24^. Hence, we also stained for the central complement pathway molecule complement C3 and found amyloid deposits to be immunoreactive also for C3 (Fig. 1h, i). These data show that in our cases cardiac ATTR amyloid did not lead to a local inflammatory response or cell death. However, we found evidence of an activation of the complement cascade as it was recently shown for ATTR amyloid affecting the carpal tunnel tissue^25^.

### Fibril extraction and analysis

The fibrils were extracted, following a protocol that was previously described. It avoids harsh chemical or denaturing conditions and allows the isolation of large quantities of structurally intact amyloid fibrils from diseased heart tissue^26^. Denaturing gel electrophoresis showed a wide band at approximately 8 to 12 kDa (Supplementary Fig. 1a). This variable mass is consistent with type A ATTR fibrils, which consists of fragmented TTR. Whereas the full-length TTR has a molecular mass of 15.8 kDa. Mass spectrometry confirmed the presence of fragmented TTR. We were able to assign seven prominent fragments within error (±0.2 Da) (Supplementary Fig. 2c). The deconvoluted mass spectra showed one major peak that we could assign to fragment 44–127. The peaks of smaller intensity were assigned to the fragments 47–127, 49–127, 50–127, 52–127, 53–127, and 58–127. The notion that most C-terminal fragments start within the position 44 to 58 has been reported previously^18,20^. Considering a cysteine carbamidomethylation (+57.02 Da) as possible modification, which has been reported previously^27,28^, we found a broad variety of fragments covering entirely or partially the N-terminal fragment. We found two peaks to which we assigned a C-terminal as well as a N-terminal fragment which extends from Pro11 to Lys35. At a monoisotopic mass of 9,034.57 Da we found the N-terminal fragment 4–86 and the C-terminal fragment 46–127. For the mass of 9,105.58 Da we assigned the N-terminal fragment 1–84 and the C-terminal fragment 45–127 (Supplementary Fig. 2c). Other N-terminal fragments were present in a great variety. We found fragments of different lengths such as the fragment 4–86 with similar start and end positions. The intensity in the raw spectrum of these individual fragments was low, but the abundance of different fragments was high (Supplementary Fig. 3).

### Cryo-EM structure of the ATTR-wt amyloid fibril

**Fig. 2:**
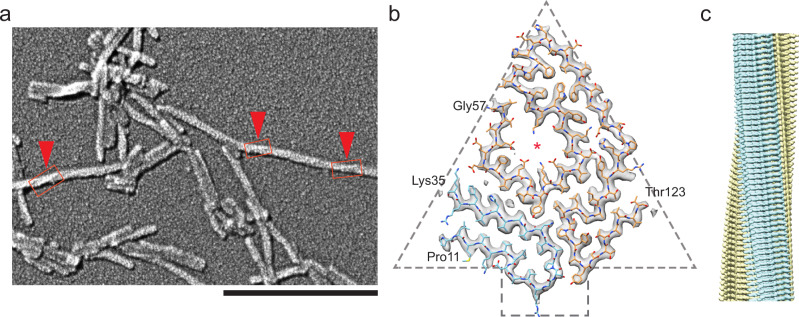
Cryo-EM reconstruction of the amyloid fibril. **a** Scanning electron microscopy image of ATTRwt fibrils after platinum side shadowing. The red markers and boxes indicate vaguely visible crossovers. Scale bar = 200 nm. **b** Cross-section of one layer of a partly transparent reconstructed 3D map superimposed with the spearhead shaped molecular model. The asterisk marks the internal cavity. **c** Side view of the reconstructed 3D map. The color coding is consistent with panel **b**.

A fibril crossover was not discernible with negative stain or cryo-EM (Supplementary Fig. 1b). 250 measurements from individual fibrils showed a mean fibril width of 10.04 ± 0.06 nm (Fig. 2a). Platinum side shadowing and scanning electron microscopy (SEM) analysis revealed relatively short fibrils that showed a barely visible crossover for some fibrils (Fig. 2a) indicating left-handedness. Extracted fibrils were prepared for cryo-EM and imaged at a 300 kV electron microscope. They appeared in the micrographs to be morphological uniform (Supplementary Fig. 1b). The images were used to pick fibril segments for three-dimensional (3D) reconstruction. Two-dimensional (2D) class averages showed a clear 4.8 Å spacing, indicating the typical cross β structure of amyloid fibrils (Supplementary Fig. 1c). A 3D map with a resolution of 2.78 Å, based on the 0.143 Fourier shell correlation (FSC) criterion (Supplementary Fig. 6), was reconstructed with the cryo-EM images (Supplementary Fig. 1b). The fibril consisted of a single twisted protofilament with a left-hand fibril twist of −1.23° and a rise of 4.82 Å (Supplementary Table 1). The density map showed two separate continuous density regions, which after structure modelling, corresponded to the residues Pro11 to Lys35 and Gly57 to Thr123, respectively. The obtained structural model had a MolProbility score of 1.32, and exhibited no Cβ, Ramachandran, or rotamer outliners (Supplementary Table 2). The N-terminus lacked resolved density for the residues Gly1-Cys10, whereas the C-terminus lacked density for residues Asn124-Glu127. Between the two regions from position Ala36 to His56, the electron density map was not resolved indicating structural movement or inhomogeneity.

### Fibril conformation

**Fig. 3:**
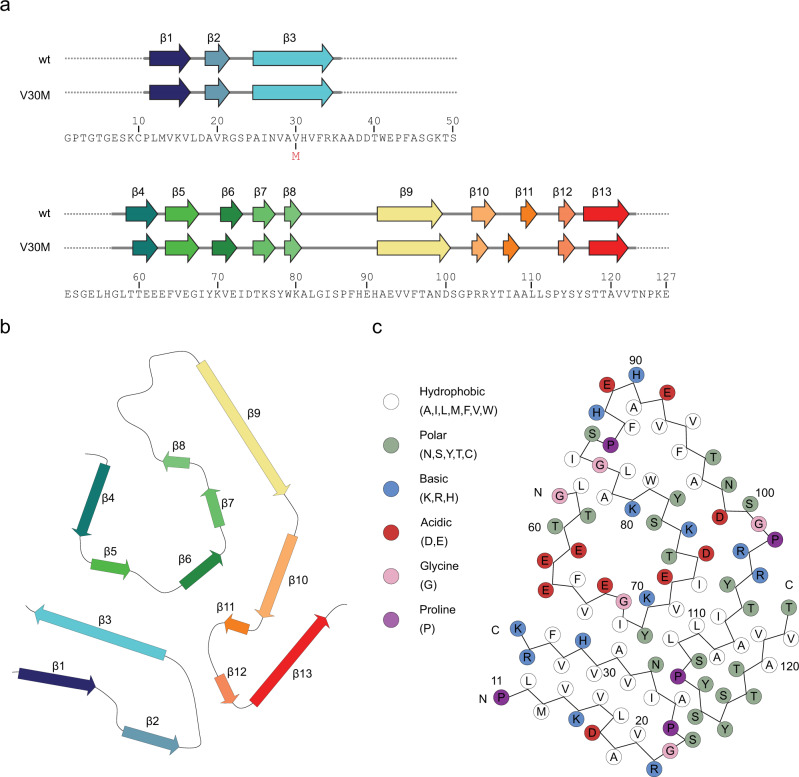
β-sheet structure of the ATTR fibril. **a** Amino acid sequence of the ATTRwt and the ATTRV30M variant. Above is a schematic representation of the secondary structure of the ATTRwt and the ATTRV30M variant from patient 2 ^23^ (Protein Data bank with entry codes: 6SDZ). Arrows indicate beta-strands, continuous lines indicate resolved structure, whereas the dotted lines indicate unresolved resolved segments. The colors correspond with (**b**) the ribbon scheme of one protein layer inside the fibril stack. **c** Protein packing schematic representation of the amino acid positions.

The cross section of the fibril resembles a spearhead shape (Fig. 2b). The fibril peptide is relatively flat and β-sheet rich. There are 13 β strands, which extend between the residues Leu12-Val16, Ala19-Arg21, Ala25-Arg34, Thr59-Glu62, Phe64-Gul66, Val71-Ile73, Thr75-Ser77, Trp79-Lys80, Glu92-Asn98, Arg103-Tyr105, Ala109-Leu110, Tyr114-Ser115, and Ser117-Val122 (Fig. 3b). Two major hydrophobic clusters are detectable. The first one is formed by six valines, two leucines, and one isoleucine residue of the N-terminal region. The second hydrophobic cluster is stabilizing the C-terminal region and is formed by residues Val71, Ile73 and Ile107, Leu110, and Leu111 (Fig. 3c). The C-terminal region of the peptide creates a probably water filed cavity, lined with polar and ionic residues Thr59 to Lys80 (Fig. 2b). The cavity is roughly 10 Å in diameter, measured using the electron density map.

The stack of protein layers is mainly stabilized by backbone-backbone hydrogen bonds of the beta sheets. Additionally, there are stabilizing interactions between side chains. Lys15 builds a salt bridge with the Asp18 (Supplementary Fig. 4a). Glu63 builds a salt bridge with the Lys35 of the n + 1 Layer, which would stabilize the first region with the second (Supplementary Fig. 4b). Thr123 builds a salt bridge with Arg104 which would stabilize the loop at the C-terminal end (Supplementary Fig. 4c). Two distinct additional density spots separated from the core peptide density can be found (Supplementary Fig. 5). One of these spots could be explained by an alternative histidine side chain conformation in the N-terminal region. The other spot between the N-terminal arch and the C-terminal segment is not assignable.

## Discussion

With an ageing population, ATTRwt amyloidosis becomes an almost endemic disease in Western countries and a significant health burden. The deposition of ATTR amyloid fibrils in the heart might progress slowly, however it significantly reduces life expectancy. We isolated ATTRwt fibrils from a case presenting with severe heart failure. The patient’s heart was explanted and showed heavily damaged heart tissue. Histology and immunohistochemistry demonstrated enormous amyloid deposits, which disrupted tissue structure. An inflammatory reaction to amyloid was scarcely observed and single myocytes did not show signs of apoptosis or necrosis. The cell death might have been induced by mechanical stress of amyloid fibrils, next to other possible mechanisms like cytotoxicity by oligomers^29^.

Here we present a 2.78 Å density map of ATTRwt fibrils, similar to the ones shown in previous studies^20,21^. Mass spectrometry confirmed that our sample is composed of type A fibrils. All C-terminal fragments found here were also the most dominant in a previous study of ATTRV30M fibrils from the heart of patient with mutant TTR (patient 1)^20^. The dominant fragment in a study of the ATTRV30M fibril obtained from the eye of a patient with hereditary ATTR amyloidosis (patient 2)^21^, with a mass of 8762,26 kDa was also found here. Some of the N-terminal fragments we found extend into part of the C-terminal region (Supplementary Fig. 3) probably arise from the full-length peptide, as truncated and full-length ATTR are usually present as a mixture in ATTRwt patients^17,18^. As previously presented, the proteolytic cleavage of the TTR peptide before fibril formation seems unlikely especially in view of the presence of the full-length peptide in ATTR patients^3^. Otherwise, the two fragments would need to stay together to from a fibril that contains both fragments, i.e., the TTR peptide needs to be cleaved locally at the site of deposition and the two resulting fragments have to escape spatial dissociation before amyloid is formed as suggested elsewhere^21^. Collectively, this rather supports the contention that amyloid formation starts with complete or partly unfolded native TTR peptides, followed by fibril formation of the full-length ATTR peptides and subsequent proteolytic cleavage at the accessible region between amino acid 44 and 58, as it was already previously presented^20^.

**Fig. 4:**
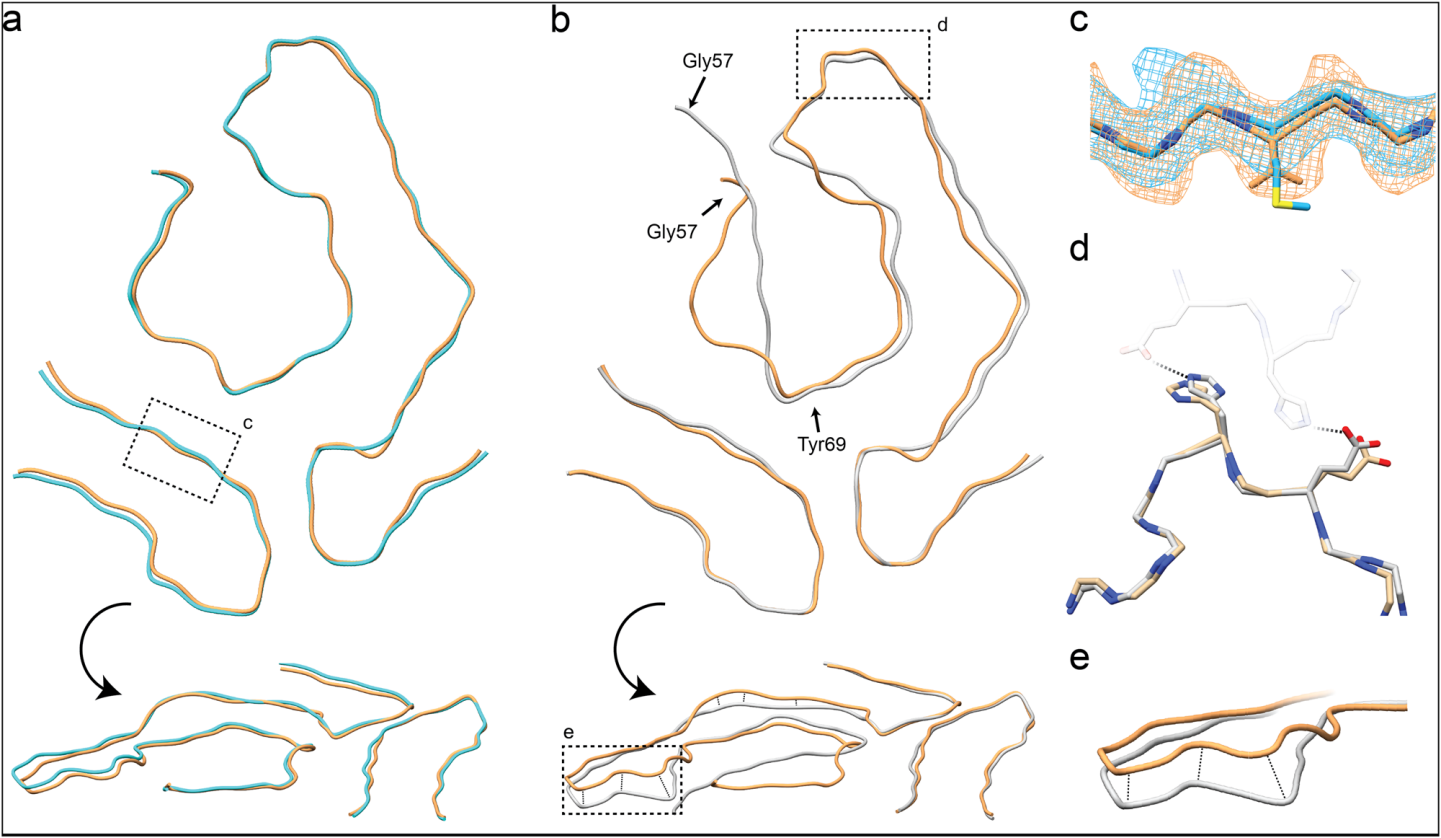
Structural comparison of the ATTRwt fibril with ATTRV30M extracted from heart and eye. **a** Top and sideview of the superimposed Ribbon diagram of the ATTRwt in yellow with the ATTRV30M from heart (patient 1) in cyan and (**b**) of the ATTRwt in yellow with the ATTRV30M (patient 2) in grey on the right. **c** Close up view comparing the density of the ATTRwt in orange and the ATTRV30M in blue from heart at position 30. The structure model shows a valine for the ATTRwt and a methionine for the ATTRV30M from heart. **d** Close up of the region of interface for the dimer of the ATTRV30M fibril from eye. One protofilament of the ATTRV30M fibril from eye (PFa), in grey compared to the same region of the ATTRwt fibril. The second protofilament (PFb) of the dimer is slightly transparent. **e** Close up of the height differences between the backbone run of the ATTRwt and ATTRV30M peptide.

We compared our ATTRwt fibril structure with the ATTRV30M fibril structures obtained from two different patients, we call patient 1 and 2 in this paper, to analyze structural similarities and differences. The ATTRV30M fibrils were also extracted from heart tissue of patient 1^20^ and for patient 2 they were extracted from the vitreous body of the eye^21^. Overall, for all three fibril structures a single fibril layer is formed by a N- and C-terminal fragment of the ATTR peptide and demonstrates a very similar spearhead like shape in their cross section (Fig. 4a, b). The resolved parts of all three reconstructions are from Pro11 to Lys35 for the N-terminal fragment and Gly57 to Thr123 for the C-terminal fragment. The N-terminal fragment is almost identical in all three cases. Especially at position 30, where the amino acid substitution is located, there is no increased density due to the methionine instead of the valine (Fig. 4c). But considering ATTRV30M amyloid fibrils can also contain wild type peptide, this lack of impact on the density would indicate the V30M variant has no major impact on the overall structure.

For the two heart derived fibril structures of the ATTRwt patient and the ATTRV30M patient 1, two from the core density separated density spots between the N-terminal and the C-terminal region could be distinguished. The spots corresponding to an alternative histidine conformation shows a weaker density, implying that this is a less common orientation (Supplementary Fig. 5). Thus, we suspect that the conformation of this histidine does not alternate with each fibril layer but has one preferred orientation and irregularly takes the alternative conformation. The occurrence of the alternative conformation could be a patient specific characteristic, which would explain why the density is smaller in the ATTRV30M reconstruction from heart of patient 1 and not present in the ATTRV30M reconstruction of patient 2. The origin of the second spot on the other hand is currently unclear. Undefined and disconnected densities may represent artefacts of the image processing but could also be signs of cofactors of the aggregation, as suggested in other amyloid studies^30^.

Comparing the C-terminal regions on their own, the ones from the two heart derived ATTR fibrils are nearly identical. The ATTRV30M fibril from patient 2 on the other hand shows regions with differences in the protein backbone height as described previously^21^. Despite identical structures of the sidechains the backbone path between residues Lys76 to Phe95 and Gly101 to Ala108 of the fibril from the ATTRV30M patient 2 differed from the backbone path of fibrils from the two heart derived fibrils with a root mean square distance of 3.3 Å between the cα atoms of the corresponding residues (Fig. 4e). These differences in backbone path seem to impact only the height of the backbone path but not the peptide arrangement in a cross-sectional view. These different protein backbone paths could be another patient specific variation of the fibril arrangement. The overall cross-sectional view of the C-terminal region of all three fibrils looks otherwise very similar. The main exception to this is the path of the C-terminal fragment from G57 to Y69. Both fibrils of ATTRwt patient and ATTRV30M patient 1 differ visibly from fibrils of ATTRV30M patient 2 (Fig. 4b). This decreases the interaction surface between the C-terminal and the N-terminal region in fibrils of ATTRV30M patient 2 and could explain the missing of previously mentioned density spots from the fibril reconstruction of ATTRV30M patient 2 (Supplementary Fig. 2a). One other major difference is, that the heart derived fibrils of ATTRwt patient and ATTRV30M patient 1 are only found as one-filament fibril. While fibrils from the study of amyloid fibrils extracted from the vitreous body of the eye of ATTRV30M patient 2 are present as single protofilament fibrils, dimers of two protofilaments and other polymeric fibrils with up to five protofilaments combined^21^. The protofilaments of the twisted dimer interact by forming hydrogen bonds between residues His90 and Glu92 of the other protofilament^21^. Superimposing the ATTR fibril structure of the ATTRwt patient and the ATTRV30M of patient 2 at the tip of the C-terminal region between residues His90 and Glu92 shows that the hydrogen bond forming amino acids do not differ significantly (Fig. 4d). It is currently unknown, why the heart derived fibrils do not form polymorphs and there does not seem be an obvious structural reason in the fibrils. But all three discussed ATTR fibrils were extracted from different patients. Thus, the small structural differences and the tendency to form polymorphs could be patient specific features. Since two samples originate from heart tissue and one from the vitreous body of the eye, we can also not exclude organ specific characteristics, that lead to these structural differences.

Evaluating all three structures, i.e., cardiac ATTRwt, cardiac ATTRV30M and ocular ATTRV30M, it is remarkable, how few overall differences are present. The small variations between fibrils from different patients point to a certain degree of freedom for some parts of fibril structure, but the overall arrangement of most amino acids and their interactions is kept intact. Hence, it is likely that the fibril morphology alone is not responsible for the variety of clinical symptoms and manifestations caused by amyloid TTR. Instead, it is reasonable to assume that various other factors play a role in the pathogenesis of ATTR amyloidosis. This is supported by the fact that the V30M point mutation of the *TTR* gene decreases the tetramer’s stability and promotes amyloid formation, therefore, an earlier onset of the disease compared to ATTRwt^31^, but has no visible impact on the fibril structure. Environmental factors, patient gender, patterns of inheritance^32,33^ and amyloid-associated components like chaperones^34^ might modulate disease manifestations.

The variation of clinical symptoms is also found in amyloid light chain (AL) amyloidosis, where AL amyloid deposits can be found in different tissues and organs^35^. But in the case of AL amyloidosis the serum free light chain concentration is very variable and each patient presents a unique light chain precursor protein, thus a unique fibril protein^36,37^. ATTR-induced amyloidosis on the other hand, shows a broad range of clinical symptoms despite the identical precursor protein and the very similar amyloid fibril structure. Currently, it is not completely understood how ATTR fibrils are related to the symptoms. Immunohistochemical (IHC) analyses demonstrated that defense mechanisms are at work in ATTR amyloidosis, presumably contributing to the devastating heart tissue damage next to mechanical stress. But the common feature of the type A fibrils from ATTR amyloidosis for these three analyzed cases is the discussed spearhead shaped cross section with a large cavity formed by N- and C-terminal ATTR peptide fragments, which could be of interest for targeted drug design and diagnosis.

## Methods

### Patient

A male patient was diagnosed with non-hereditary ATTR amyloidosis at the age of 57 years. The 4 exons of the transthyretin gene were sequenced from the blood of the patient to identify amyloidogenic variants and no variants were found in our patient. Heart failure worsened the next years and the patient received heart transplant 5 years later at an age of 62 years. Until heart transplantation the patient was not treated with disease-specific drugs. Stage of disease at the diagnosis was stage 1^38^ which corresponds to the New York Heart Association (NYHA) Functional Classification class I and was stage 3/NYHA class III at transplant. The study was approved by the ethical committees of the University of Heidelberg (S-123/2006). Informed consent for publication of details was obtained from the patient, as well as consent that these samples are used for scientific research.

### Histology and immunohistochemistry

Part of the heart explant was fixed in formalin and embedded in paraffin (FFPE), and used for histology and IHC. According to previously published protocols, the FFPE sample was cut into serial tissue sections using a microtome and dried at 55 °C for 30 min. Tissue sections were forwarded to Tissue Tek Prisma (Sakura, Umkirch, Deutschland) for deparaffinization, rehydration, Congo red and hematoxylin eosin (HE) staining. Immunohistochemistry (IHC) was carried out with commercially available monoclonal mouse antibodies directed against complement C3 (C3; clone 755, 1:400, Abcam, Berlin, Germany), complement component C9 (C9; clone 64E9, 1:400; LifeSpan Biosciences, Seattle, USA), CD3 (clone LN10, 1:100, Leica Biosystems, Wetzlar, Germany), CD68 (clone S-14M12, 1:100, Leica Biosystems, Wetzlar, Germany), a rabbit polyclonal anti-human-MPO antibody (1:2000; Dako, Carpinteria, CA, USA), a monoclonal rabbit antibody directed against caspase 3 (clone 5A1E, 1:100; Cell Signaling, Danvers, MA, USA), and non-commercially available polyclonal rabbit antibody directed against TTR (1:2000, Pineda^39^). According to the manufacturer’s instructions, FFPE sections were immunostained with the Bond Max Leica (Leica Biosystems, Wetzlar, Germany) immunostainer using the Bond Polymer Refine Detection Kit (C3, C9, CD3, CD68, MPO, caspase 3) and Bond Polymer Refine Red Detection Kit (TTR; both Leica Microsystems, Wetzlar, Germany). For Antigen retrieval, Leica ER1-Bond Epitope Retrieval Solution 1 (C3, C9, CD3) or Leica ER2-Bond Epitope Retrieval Solution 2 (TTR, caspase 3, CD68, MPO) were used.

### Digital image analysis

Digital image analysis assessed the amyloid load of Congo red-stained tissue sections, as previously described^25^. In brief, Hamamatsu NanoZoomer 2.0 RS scanner (Hamamatsu Photonics Deutschland GmbH, Herrsching am Ammersee, Germany) scanned sections at 400 times magnification in sequential bright field and fluorescence mode (excitation/emission wavelength 560/607 nm for detection of Congo red fluorescence and 485/526 nm for compensation of unspecific background signal, respectively). ImageJ version 1.52p (National Institutes of Health, USA) selected and counted all tissue pixels in the bright field image and all pixels showing Congo red signal in the fluorescence image (compensated for unspecific background), respectively. The ratio of both pixel counts gives the amyloid load.

### Fibril extraction

Fibrils were extracted from the amyloidotic tissue according to a recently described extraction protocol^26^. In brief, 125 mg of frozen human heart tissue was diced with a scalpel and washed with 500 µL ice cold Tris-calcium buffer [20 mM Tris, 138 mM NaCl, 2 mM CaCl_2_, 0.1% (w/v) NaN_3_, pH 8.0]. The sample was centrifuged for 5 min at 3100 × *g* and 4 °C. The supernatant was removed, it did not show amyloid fibrils with negative stain EM. The pellet was resuspended in 500 µL ice-cold Tris-calcium buffer and homogenized again with a Kontes Pellet Pestle. This procedure was repeated four more times. After the last step, the pellet was resuspended in a 1 mL solution of freshly prepared 5 mg/mL *Clostridium histolyticum* collagenase (Sigma) in Tris calcium buffer with ethylenediaminetetraacetic acid (EDTA)-free protease inhibitor (Roche). After incubation overnight on a shaker at 37 °C the tissue material was centrifuged for 30 min at 3100 × *g* and 4 °C. The supernatant was removed and showed some fibril bundles, which looked similar to fibrils from the final washing steps with negative stain EM. The pellet was resuspended in 500 µL Tris EDTA buffer [20 mM Tris, 140 mM NaCl,10 mM EDTA, 0.1% (w/v) NaN_3_, pH 8.0], and centrifuged for 5 min at 3100 × *g* and 4 °C. This washing step was repeated two more times. Afterwards the tissue pellet was homogenized in 100 µL ice-cold water and centrifuged for 5 min at 3100 × *g* at 4 °C. The supernatant containing the water-soluble fibrils was retained. This extracting step was repeated five more times.

### Platinum side shadowing and scanning electron microscopy

For SEM, formvar/Carbon 200 mesh cupper grids (electron microscopy sciences) were glow-discharged at 20 mA for 40 s. A 3.5 µL aliquot of ATTRwt fibril containing watery solution was applied to the grid. The excess was blotted with filter paper. The grids were then dried at room temperature. A 1 nm thick layer of platinum was evaporated from an angle of 30° onto the grid with a Blazers TKR 010. The grids were inspected using a Hitachi S-5200 scanning electron microscope (Hitachi) at 10 kV acceleration voltage.

### Cryo-EM sample preparation and data collection

The quality of grids was regularly checked at a 2100 F transmission electron microscope (Joel), operating at 200 kV. Grids were prepared with an automatic plunge freezer EM GP2 (Leica). For data collection a C-flat 1.2/1.3 400 mash holey carbon cupper grid was glow-discharged at 20 mA for 40 s. A 3 µL aliquot of ATTRwt fibril containing watery solution was applied to the grid. The excess was blotted with filter paper for 4 sec with a temperature of 21 °C and a relative humidity of 90% as surrounding conditions. After blotting the grid was plunge frozen in liquid ethane. Electron micrographs were collected on a Titan Krios transmission electron microscope (Thermo Fisher) with a K2-summit detector in counting mode, at 300 kV and a magnification of 130,000× (further details in Supplementary Table 1). The software SerialEM v3.9 was used for data collection.

### Reconstruction of the 3D map

The raw data movie frames were gain-corrected with IMOD^40^. Motion correction was done with the own implementation function in RELION 3.1^41^. CTF estimation was done using CTFfind 4.1^42^. All subsequent image-processing steps were performed using helical reconstruction methods in RELION^43,44^. The start and end coordinates of fibrils were manually picked and particles were extracted with a box size of 300 pix and an inter box distance of 10% of the box length. A reference free 2D classification with 50 classes and a regularization parameter of 2 followed. 2D classes without visible fibril outliners were discarded. A featureless cylinder was used as an initial model for the initial 3D classification. This generated a primary model which was used for further classification. The classification was done with 5 classes and a T value of 5. Three classes showed a well resolved structure and were selected manually. The particles from those selected classes were reconstructed with local optimization of helical parameters using auto-refinement. Post-processing yielded a map with an estimated resolution of 2.94 Å. CTF-refinement^45^ and Bayesian polishing^41^ further improved the resolution of the map to the final resolution of 2.78 Å. The final post-processing with a soft edged mask and an estimated map sharpening B-Factor of −76.54 yielded a model with a twist of −1.234° and a helical rise of 4.82 Å. The resolution was estimated with the FSC curve of two independently refined half maps at 0.142 (FSC gold standard).

### Model building and refinement

The model of the ATTRV30M variant was altered using Chimera^46^. The Methionine at position 30 was changed to a Valine. The phenix.dock_in_map function in Phenix^47^ was used to superimpose the model with the map, followed by manual building in Coot^48^. The resulting model was refined by real space refinement using the phenix.real_space_refine function. Non-crystallographic symmetry and secondary structure restraints were imposed. The quality of the model was assessed using Molprobity^49^.

### Mass spectrometry

Samples were extracted from patient tissue as described earlier. 500 µl of the water extract were lyophilized and subsequently resuspended and incubated in 6 M Guanidine Hydrochloride (Gnd-HCl) over-night. The sample was adjusted to 15 µl using 0.1% (v/v) trifluoroacetic acid (TFA). Because the sample was diluted in TFA, the guanidine concentration was reduced and the salt could be removed by liquid chromatography alone, so no further desalting was required. Samples were separated by liquid chromatography using a U3000 RSLCnano (Thermo Fisher Scientific) online coupled to the mass spectrometer with an Acclaim® PepMapTM analytical column (75 µm × 500 mm, 2 µm, 100 Å pore size; Thermo Fisher Scientific) in combination with a C18 µ-precolumn (0.3 mm × 5 mm; PepMap, Dionex LC Packings; Thermo Fisher Scientific). First, samples were washed with 0.1% (v/v) TFA for 5 min at a flow rate of 30 µL/min. Subsequent separation was carried out employing a flow rate of 250 nL/min using a gradient consisting of solvent A [0.1% (v/v) formic acid] and solvent B [86% (v/v) ACN, 0.1% (v/v) formic acid]. The main column was initially equilibrated in a mixture containing 5% (v/v) solvent B and 95% (v/v) solvent A. For elution, the percentage of solvent B was raised from 5 to 15% over a period of 10 min, followed by an increase from 15 to 40% over 20 min. Fractions from the main column directly eluted into the ionization module and were further analyzed by mass spectrometry. Samples were measured using an LTQ Orbitrap Elite system (Thermo Fisher Scientific). The mass spectrometer was equipped with a nanoelectrospray ion source and distal coated SilicaTips (FS360-20-10-D, New Objective). The instrument was externally calibrated using standard compounds (LTQ Velos ESI Positive Ion Calibration Solution, Pierce, Thermo Scientific). The system was operated using the following parameters: spray voltage, 1.5 kV; capillary temperature, 250 °C; S-Lens radio frequency level, 68.9%. The software XCalibur 2.2 SP1.48 (Thermo Fisher Scientific) was used for data-dependent MS/MS analyses. Full scans ranging from mass to charge ratio (m/z) 370 to 1700 were acquired in the Orbitrap at a resolution of 30,000 (at m/z 400) with automatic gain control enabled and set to 10^6 ^ions and a maximum fill time of 500 ms. MS/MS scans were performed in the quadrupole at normal scan speed.

The raw data was deconvoluted by the MASH Explorer 52 using default settings and the “Quick Deconvolution” feature. All calculated monoisotopic masses with a score equal or above 90% resulting from initial m/z peaks with 1 charge or more were considered as correct and are shown in the mass spectrum of this work. Masses were manually assigned using the software mMass v5.5.0^50^

### Reporting summary

Further information on research design is available in the Nature Research Reporting Summary linked to this article.

## Supplementary information

Supplementary Information

Reporting Summary

## Data Availability

The reconstructed cryo-EM map was deposited in the Electron Microscopy Data Bank with the accession code EMD-15361. The coordinates of the fitted atomic model were deposited in the Protein Data Bank under the accession code 8ADE [10.2210/pdb8ADE/pdb]. The data that support the findings of this study are available from the corresponding authors upon reasonable request. The source data underlying Supplementary Fig. 1a, 2c and 4 are provided as a Source Data file. Source data are provided with this paper.

## Source data

Source Data
