# Supplementary material for: Cryo-EM structure of an ATTRwt amyloid fibril from systemic non-hereditary transthyretin amyloidosis
Source: Nat Commun. 2022 Oct 27;13:6398. doi: 10.1038/s41467-022-33591-4 (PMC9613903; doi:10.1038/s41467-022-33591-4)
Supplement: Supplementary file 1 — Supplementary Information [file 41467_2022_33591_MOESM1_ESM.pdf]

Supplementary Information

Cryo-EM structure of an ATTRwt amyloid fibril from systemic non-hereditary transthyretin amyloidosis

Maximilian Steinebrei[1], Juliane Gottwald[2], Julian Baur[1], Christoph Röcken[2], Ute Hegenbart[3], Stefan O. Schönland[3], Matthias Schmidt[1]

Affiliations:

1. Institute of Protein Biochemistry, Ulm University, Helmholtzstrasse 8/1, Ulm D-89081, Germany
2. Department of Pathology, Christian-Albrechts-University Kiel, University Hospital Schleswig-Holstein, Arnold-Heller-Str. 3, Building U33, Kiel D-24105, Germany
3. Medical Department V, Amyloidosis Center, Heidelberg, University Hospital Heidelberg, Im Neuenheimer Feld 400, Heidelberg D-69120, Germany.

***Correspondence should be addressed to:**
Matthias Schmidt; matthias.schmidt@uni-ulm.de

Supplementary figure 1

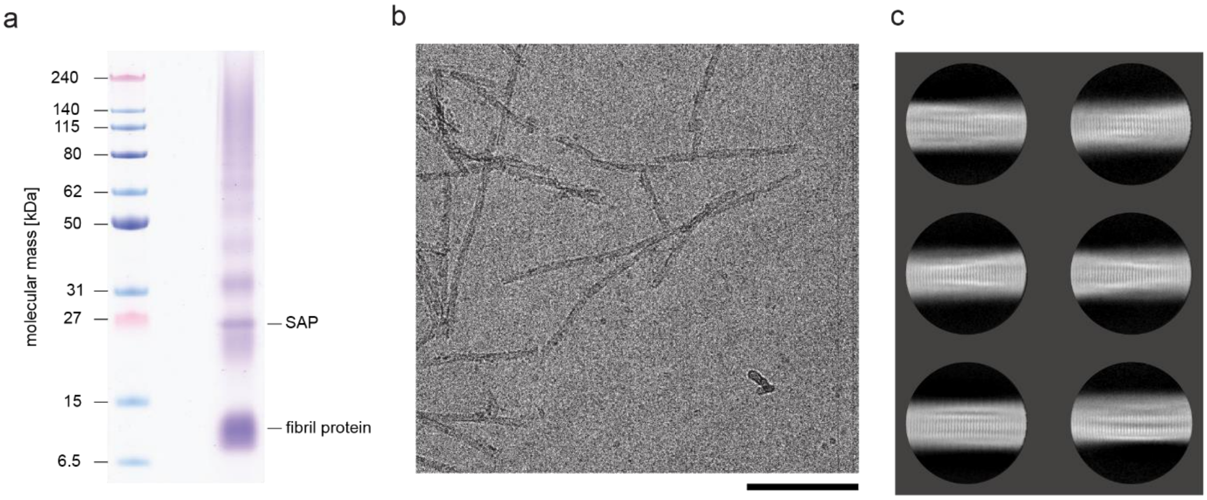

Supplementary figure 1

Characteristics of ATTRwt fibril extraction from heart

(a) Coomassie-stained denaturing electrophoresis gel of the extracted ATTRwt fibrils from heart tissue. The first lane shows the marker. The fibril proteins and serum amyloid P component (SAP) are indicated in the Figure. Three independent experiments showed similar results. (b) One representative cryo-EM image of the dataset used for the reconstruction of the shown ATTRwt amyloid fibrils from ATTRwt amyloidosis. Scale bar: 100 nm. (c) 2D class averages showing the protein stacks of the ATTRwt amyloid fibril. Source data are provided as a Source Data file for (a).

Supplementary figure 2

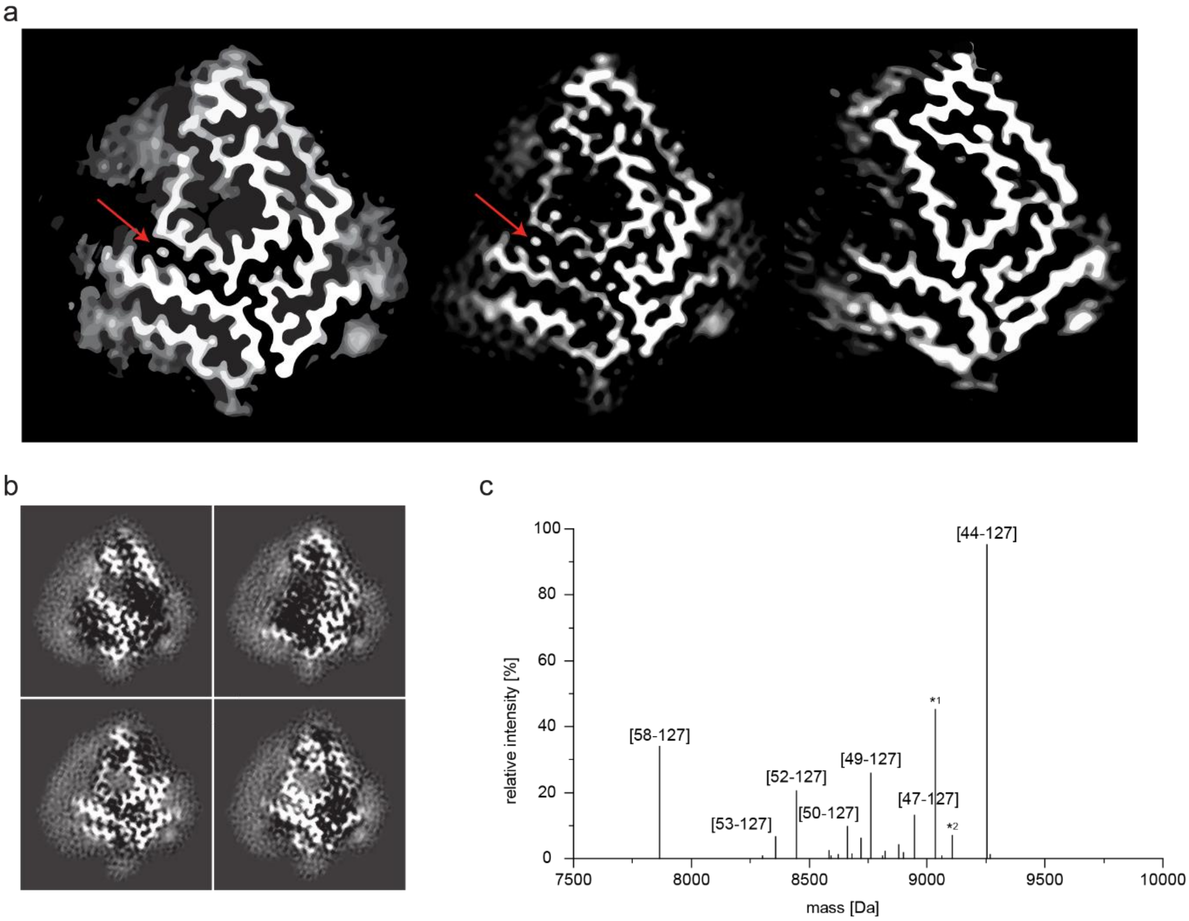

Supplementary figure 2

Fibril characteristics of ATTR reconstructions

(a) 5 Å thick cross-sectional view of the 3D density map of the ATTRwt fibril on the left, the ATTRV30M of patient 1 from heart in the middle and the ATTRV30M of patient 2 from eye on the right. The red arrows indicate undefined density spots which are lacking in the ATTRV30M from patient 2. (b) Four cross-sectional views of the 3D density map of the ATTRwt fibril, each view showing a 1 Å thick slice. (c) Deconvoluted mass spectra between the range of 7.5 kDa and 10 kDa with assigned fragments. The peak with monoisotopic mass of 9034,57 Da (*1) could be assigned to a fragment 4-86 or 46-127 and the peak with monoisotopic mass of 9105,58 Da (*2) could be assigned to a fragment 1-84 or 45-127. Source data are provided as a Source Data file for (c).

Supplementary figure 3

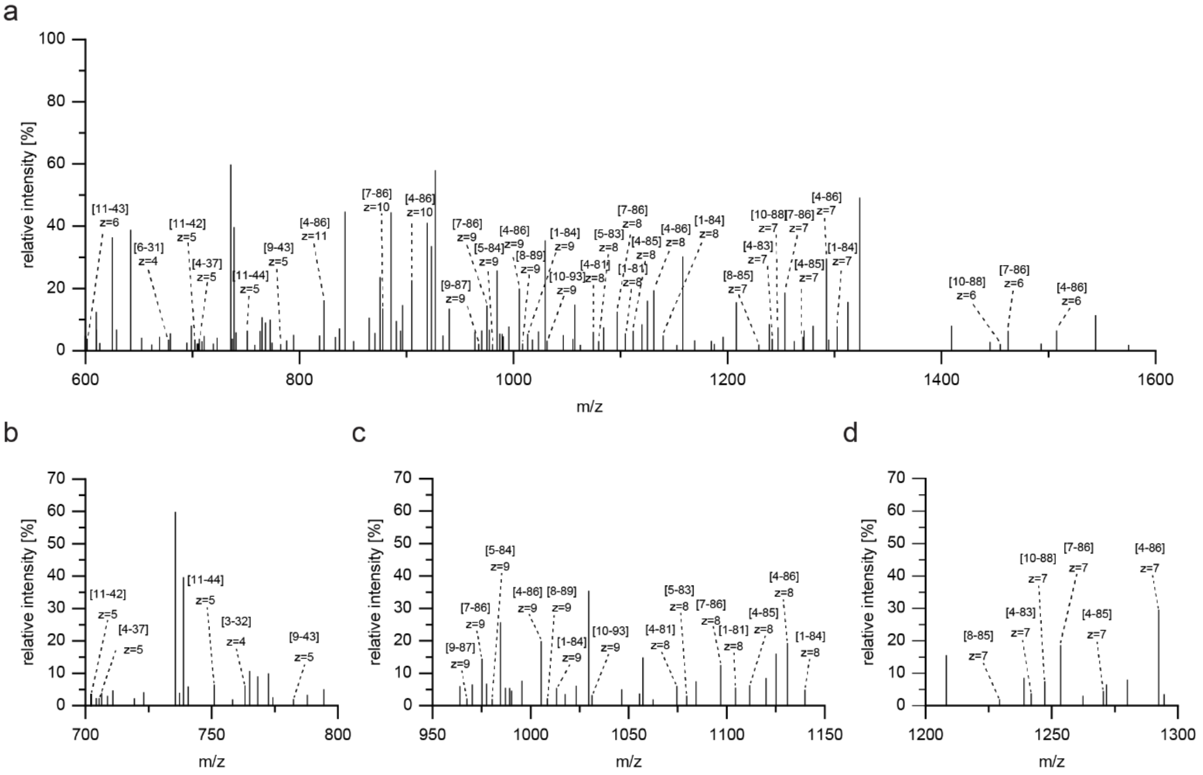

Supplementary figure 3

Raw mass spectrometry analysis of ATTR sample.

(a) Full mass spectrum showing a range of 550-1600 m/z of the fibril solution as sum of all MS spectra recorded during elution. The spectrum depicts the high abundance of different fragments, that cover the N-terminal region. For a better overview only N-terminal fragments were labelled. Zoomed areas of the full mass spectrum between the range 700 -800 m/z (b), the range of 950-1150 m/z (c) and the range of 1200-1300 m/z (d). Source data are provided as a Source Data file.

Supplementary figure 4

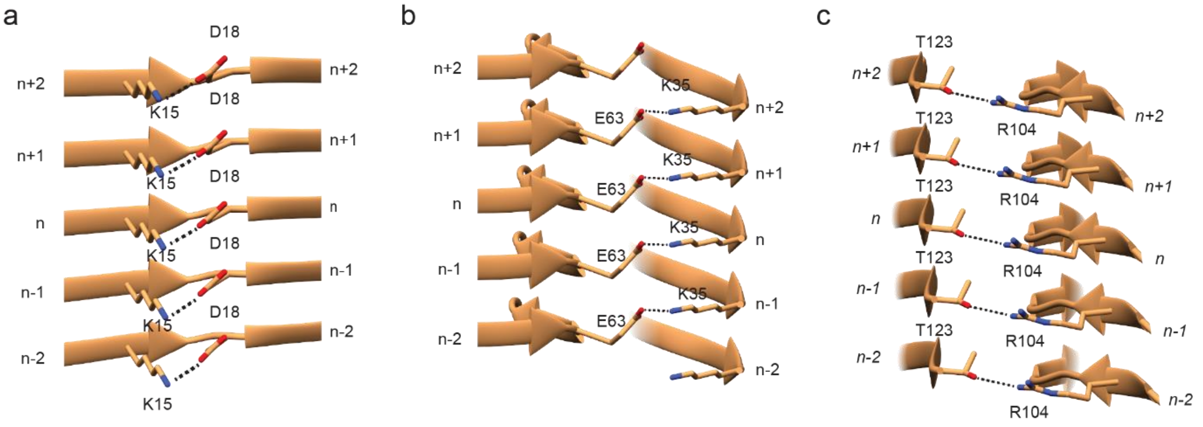

Supplementary figure 4

Stabilizing salt bridges within fibril stacks

Stack of five fibril protein layers showing the stabilizing salt bridge between Lys15 and Asp18 in (a), between Lys35 of layer n and Glu63 of the layer n-1 in (b) and between Arg104 and Thr123 (c).

Supplementary figure 5

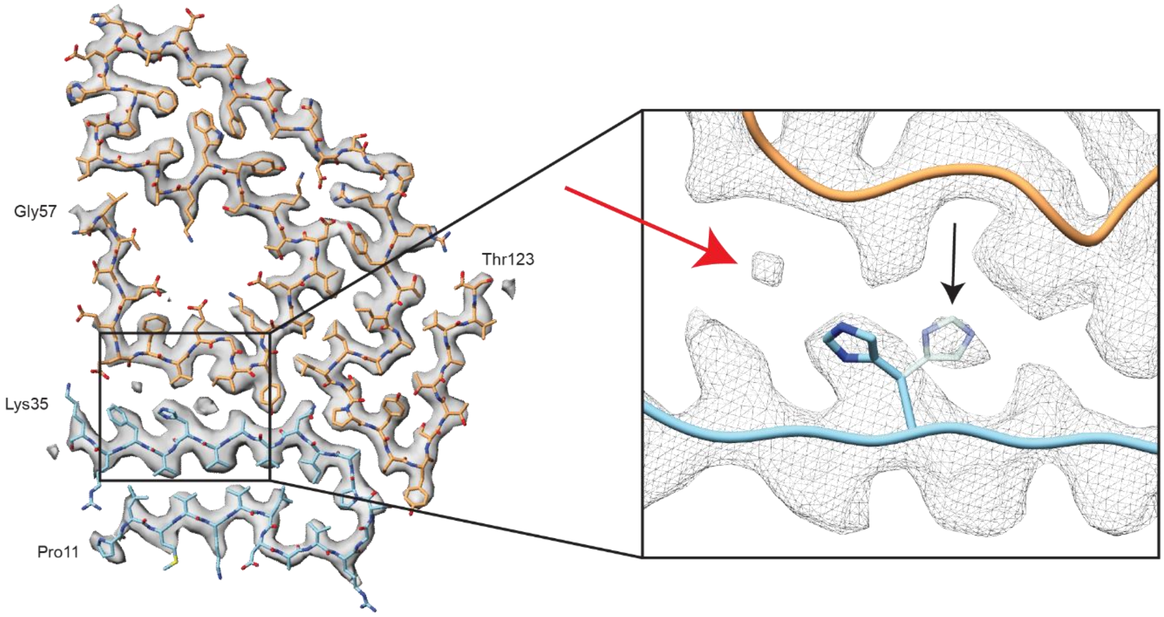

Supplementary figure 5

Alternative conformation of the histidine 31

The black arrow points to the extra density surrounding position 31, which revealed an alternative conformation for the adjacent histidine. The red arrow points to the free density spot, which could not be assigned, but it could represent artefacts of the image processing or a cofactor of the aggregation.

Supplementary figure 6

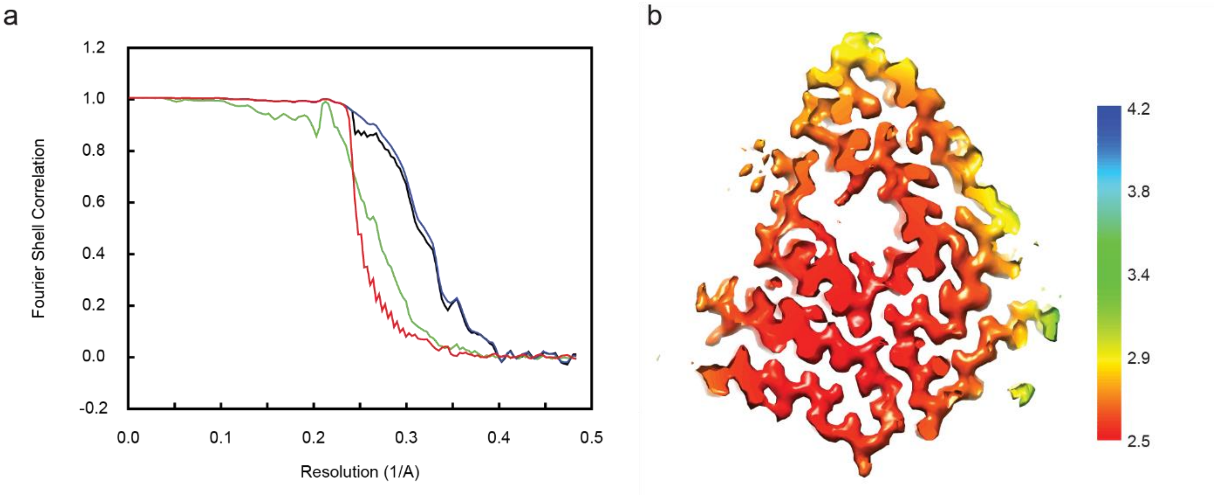

Supplementary figure 6

Reconstruction of ATTRwt amyloid fibril

(a) FSC between two half-maps (black line = FSC corrected, green line = FSC unmasked maps, blue line FSC masked maps, red line = corrected FSC phase randomized masked maps). (b) Cryo-EM map of the ATTRwt fibril with estimation of local resolution. Source data are provided as a Source Data file for (a).

Supplementary Tables

Supplementary Table 1

Microscope	Titan Krios (Thermo Fisher Scientific)
Camera	K2 Summit (Gatan)
Acceleration voltage (kV)	300
Magnification	x 130,000
Defocus range (µm)	-1.2 to -2.5
Dose rate (e^-/Å/s)	~3
Number of movie frames	40
Exposure time (s)	12
Total electron dose (e^-/Å)	44.9
Pixel size (Å)	1.04
Box size (pixel)	300
Inter box distance (Å)	31.2
Number of extracted segments	117,516
Number of segments after 2D classification	115,511
Number of segments after 3D classification	87,666
Resolution, 0.143 FSC criterion (Å)	2.78
Map sharpening B-Factor ($Å^2$)	-76.54
Helical rise (Å)	4.82
Helical twist (°)	-1.234

Supplementary Table 1

Data collection and reconstruction parameters.

Supplementary Table 2

Model resolution (Å)	
FSC threshold 0.143	2.78
FSC threshold 0.5	3.1
Map sharpening B factor (Å2)	-47.77
Model composition	
Non-hydrogen atoms	50236
Protein residues	644
Ligands	-
B factors (Å2)	
Protein	64.33
Ligand	-
R.m.s. deviations	
Bond lengths (Å)	0.012
Bond angles (°)	1.537
Validation	
MolProbity score	1.32
Clashscore	3.08
Poor rotamers (%)	0
Ramachandran plot	
Favored (%)	96.59
Allowed (%)	3.41
Disallowed (%)	0

Supplementary Table 2

Modelling parameters.
